# IRGC1, a testis-enriched immunity related GTPase, is important for fibrous sheath integrity and sperm motility in mice

**DOI:** 10.1016/j.ydbio.2022.05.011

**Published:** 2022-08

**Authors:** Yuki Kaneda, Haruhiko Miyata, Keisuke Shimada, Yuki Oyama, Rie Iida-Norita, Masahito Ikawa

**Affiliations:** a Research Institute for Microbial Diseases, Osaka University, 3-1 Yamadaoka, Suita, Osaka, 5650871, Japan; b Graduate School of Pharmaceutical Sciences, Osaka University, 1-6 Yamadaoka, Suita, Osaka, 5650871, Japan; c The Institute of Medical Science, The University of Tokyo, 4-6-1 Shirokanedai, Minato-ku, Tokyo, 1088639, Japan; d Center for Infectious Disease Education and Research, Osaka University, 2-8 Yamadaoka, Suita, Osaka, 5650871, Japan

**Keywords:** CRISPR/Cas9, Fibrous sheath, Flagellum, p47 GTPases, Sperm motility

## Abstract

Immunity-related GTPases (IRGs), also known as p47 GTPases, are a family of interferon-inducible proteins that play roles in immunity defense against intracellular pathogens. Although the molecular functions of IRGs have been well studied, the function of the family member, IRGC1, remains unclear. IRGC1 is unique among IRGs because its expression is not induced by interferon and it is expressed predominantly in the testis. Further, IRGC1 is well conserved in mammals unlike other IRGs. Here, we knocked out (KO) *Irgc1* in mice using the CRISPR/Cas9 system and found that the fertility of *Irgc1* KO males was severely impaired because of abnormal sperm motility. Further analyses with a transmission electron microscope revealed that the fibrous sheath (FS), an accessory structure of the sperm tail, was disorganized in *Irgc1* KO mice. In addition, IRGC1 was detected in the sperm tail and fractionated with FS proteins. These results suggest that IRGC1 is a component of the FS and is involved in the correct formation of the FS.

## Highlights

• IRGC1, a testis-enriched immunity related GTPase, is important for male fertility in mice.
• *Irgc1* KO mice exhibited impaired sperm motility.
• Fibrous sheath structures were disrupted in *Irgc1* KO spermatozoa.
• IRGC1 fractionates with fibrous sheath proteins, suggesting that IRGC1 is a component of the fibrous sheath.

## Graphical abstract

**Figure:**
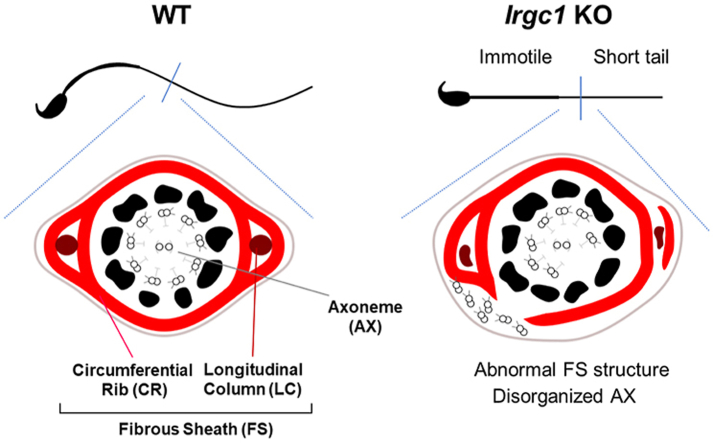

## 1 Introduction

Infertility is one of the most prevailing medical problem in the world and the prevalence of infertility is one out of every six couples of reproductive age (Agarwal et al., 2019). The distribution of infertility due to male factors varies between the regions of the world and is estimated to range from 20% to 70% (Agarwal et al., 2015). Male infertility can be caused by no or low sperm production (azoospermia or oligospermia, respectively), abnormal sperm morphology (teratospermia), and/or abnormal sperm motility (asthenospermia); however, most of the causes of abnormal sperm formation and/or function remain unclear.

The flagellum is a motility apparatus of the spermatozoa and can be divided into three parts, the midpiece where mitochondria are helically arranged around the outer dense fibers (ODFs) and axoneme, the principal piece that contains the fibrous sheath (FS) surrounding the ODFs and axoneme, and the end piece without accessory structures (Eddy et al., 2003). The FS consists of the longitudinal columns (LCs) that are localized adjacent to the doublets 3 and 8 of the axoneme and the rib that connects the two LCs. The FS serves not only as a structural component that provides elastic rigidity for sperm motility but also as a scaffold for several glycolytic and signaling molecules (Krisfalusi et al., 2006). Molecular components of the FS have been studied and A-kinase anchoring protein 3 (AKAP3) and 4 (AKAP4) have been identified as primary components of the FS (Fiedler et al., 2013). Abnormal formation of the FS could result in impaired sperm motility and male infertility. This condition is called a dysplasia of the fibrous sheath, which is currently recognized as morphological abnormalities of the sperm flagella (Nsota Mbango et al., 2019).

Immunity-related GTPases (IRGs) were first identified from their expression being induced strongly by interferon γ (IFNγ) (Taylor et al., 1996; Boehm et al., 1998) due to the presence of interferon-stimulated response elements (ISRE) and/or γ-activated sequence sites (GAS) in their promoter regions (Bekpen et al., 2005). Several IRGs have been reported to have critical functions in pathogen resistance in mice (Hunn et al., 2011). For instance, *Irgm1*, *Irgm3,* and *Irgd* KO mice exhibit significant susceptibility to the infection of a number of pathogens and there are functional distinctions among these genes (Taylor et al., 2007). Among the IRGs, *Irgc1* is a clear exception in the family in terms of lacking both ISRE and GAS elements and its expression restricted to the testis (Bekpen et al., 2005); however, its function remains to be determined.

Here, we generated *Irgc1* KO mice using the CRISPR/Cas9 system to analyze its function *in vivo*. *Irgc1* KO mice exhibit abnormal FS morphology, impaired sperm motility, and subfertility, suggesting that IRGC1 plays roles in the correct formation of the FS.

## 2 Results

### 2.1 *Irgc1* is expressed predominantly in the testis

**Fig. 1:**
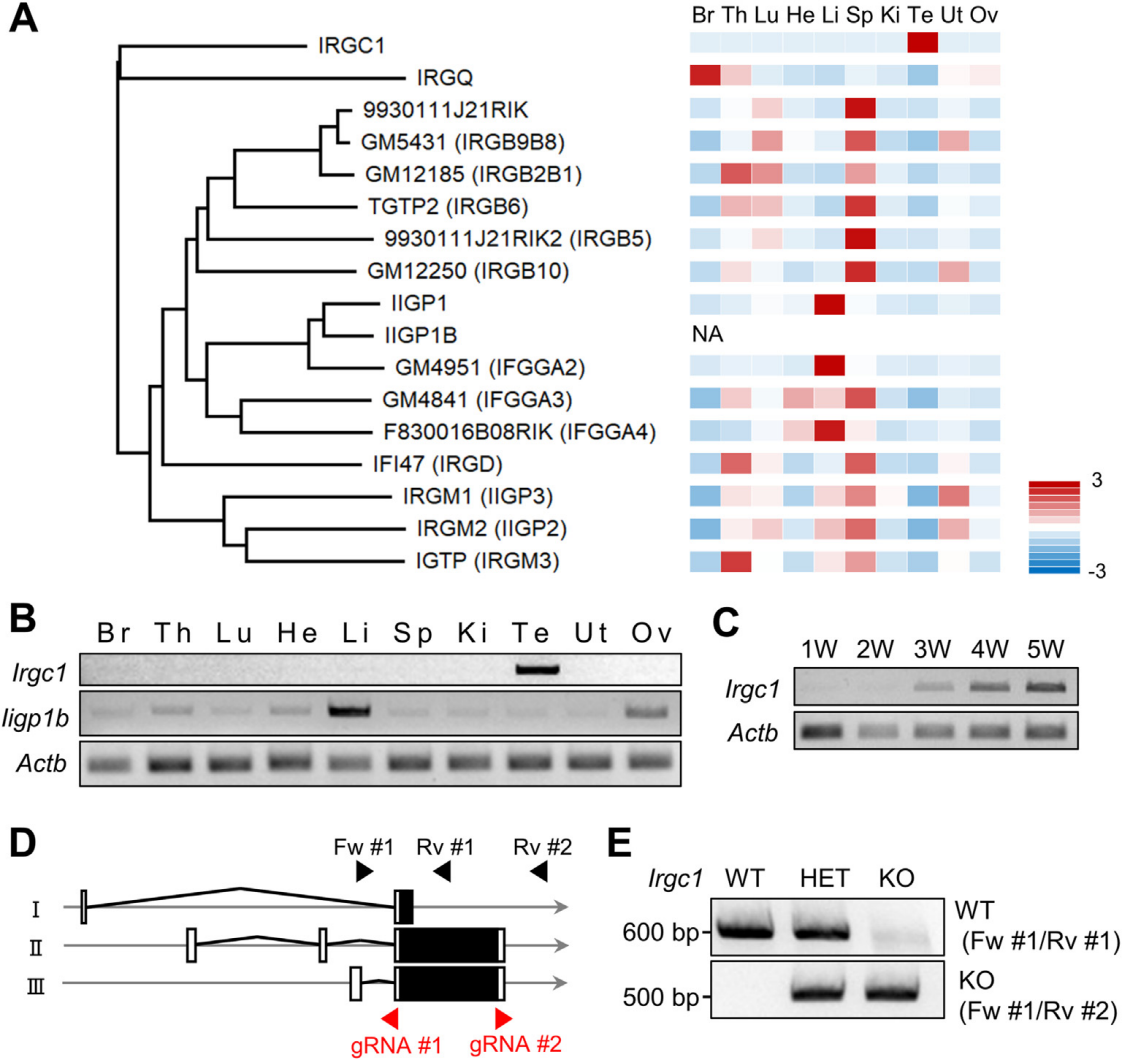
*Irgc1* is a testis-enriched gene. (A) Phylogenetic analyses of IRGC paralogues. Z-scores of averaged gene expression values across mouse adult tissues (Li et al., 2017) are shown on the right. There are no RNA-seq data about *Iigp1b*. *Irgc1* is expressed predominantly in the testis. Br: brain, Th: thymus, Lu: lung, He: heart, Li: liver, Sp: spleen, Ki: kidney, Te: testis, Ut: uterus, and Ov: ovary. (B) RT-PCR of *Irgc1* and *Iigp1b* in mouse adult tissues. *Actb* as control. (C) RT-PCR of *Irgc1* using RNAs obtained from mouse at various postnatal testes. *Actb* as control. (D) CRISPR/Cas9 targeting scheme. White boxes indicate untranslated regions while black boxes indicate protein coding regions. There are three variants in *Irgc1*. (E) Genotyping of *Irgc1* mutant mice. Fw #1-Rv #1 and Fw #1-Rv #2 primers in Fig. 1D were used.

By performing Protein Blast (https://blast.ncbi.nlm.nih.gov/Blast.cgi) analysis of mouse IRGC1, other IRGs that have similar sequences to IRGC1 were identified. The phylogenetic relationship of these proteins is shown in Fig. 1A. Despite *Irgc1* having close relationships with other *Irgs*, expression of mouse *Irgc1* and its human ortholog, *IRGC*, is not induced by interferon (Bekpen et al., 2005). IRGs exhibit higher sequence diversity within and between species (Hunn et al., 2011), with the only exception being IRGC1, which is highly conserved between humans and mice (Fig. S1A). Further, *Irgc1*, but not other *Irgs*, is expressed predominantly in the mouse testis (Fig. 1A). We performed RT-PCR using multiple tissues of adult mice and confirmed that *Irgc1* is expressed predominantly in the testis (Fig. 1B). The first cycle of spermatogenesis in mouse seminiferous tubules completes within the first 35 days after birth (Kluin et al., 1982). To investigate temporal expression of *Irgc1* in mouse spermatogenesis, we conducted RT-PCR using postnatal testes of mice from different ages. This analysis reveals that *Irgc1* is expressed from around postnatal three weeks, when round spermatids are observed (Fig. 1C). By analyzing the single RNA sequence database (Hermann et al., 2018), we confirmed *Irgc1* expression in spermatids in both mice and humans (Figs. S1B and C). These results suggest that IRGC1 may play roles in late spermatogenesis and/or fertilization.

### 2.2 Generation of *Irgc1* knockout mice

To uncover the function of *Irgc1 in vivo*, we generated *Irgc1* KO mice using the CRISPR/Cas9 system. Using an *in silico* search (www.ensembl.org), we found that *Irgc1* has three splice variants. Two guide RNAs (gRNAs) were designed to remove the entire open reading frame of these variants (Fig. 1D). Ribonucleoprotein complex containing the gRNA and CAS9 was then electroporated into 50 pronuclear embryos and 40 two-cell embryos were transferred into the oviducts of pseudopregnant females. Twelve of the 17 pups had large deletions, and one was caged with two wild-type (WT) females to obtain the F1 generation. Subsequent mating resulted in a KO mouse with a 1266 bp deletion (Fig. S1D), which was confirmed by PCR (Fig. 1E). No overt abnormalities were found in the KO mice.

### 2.3 Fertility of *Irgc1* KO males is severely impaired

**Fig. 2:**
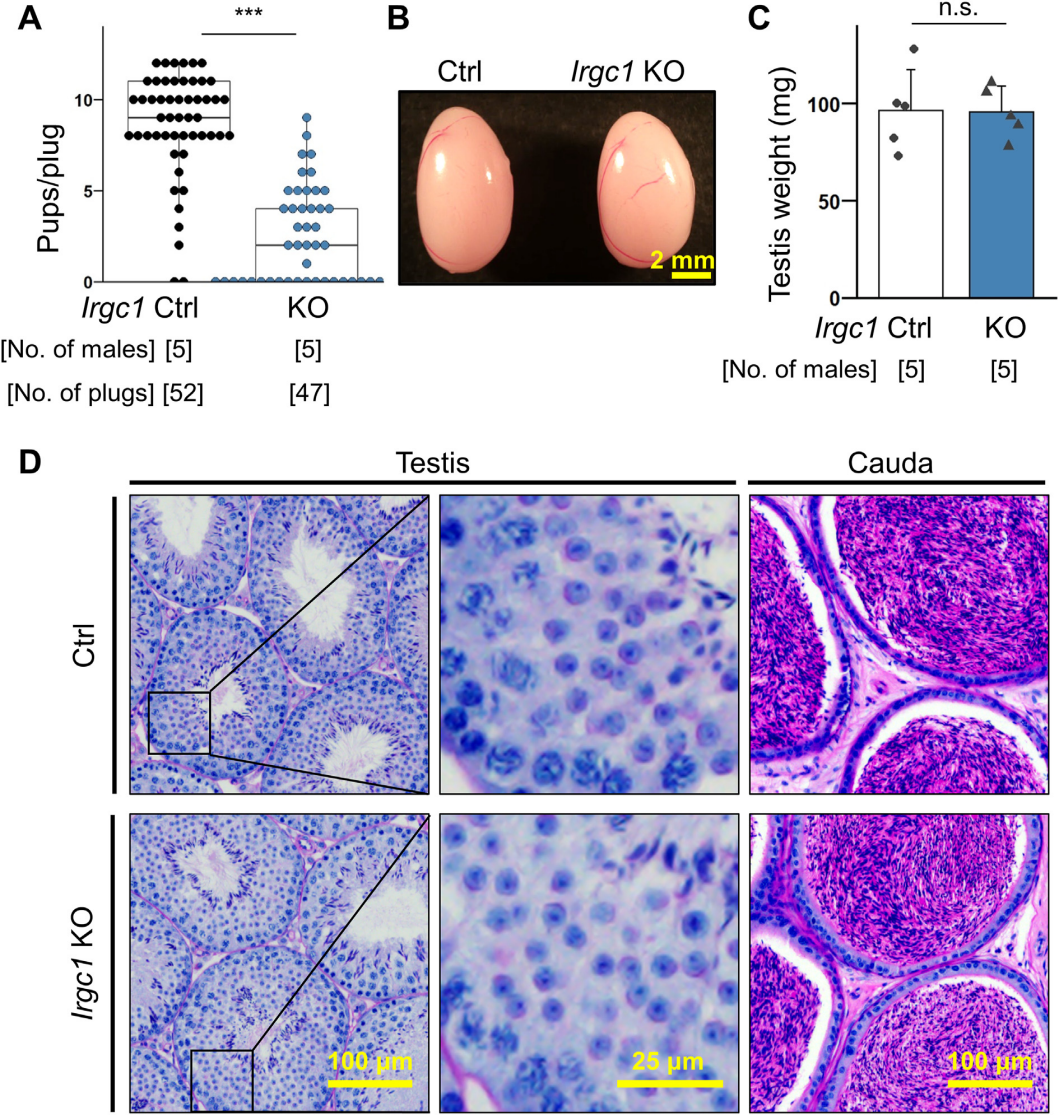
IRGC1 is important for male fertility. (A) Number of pups born per plug detected. Three WT females were caged with each male for three months. Average pups/plug was Ctrl ​= ​8.65 ​± ​2.88 pups/plug; *Irgc1* KO ​= ​2.38 ​± ​2.63 pups/plug. *P* ​= ​1.88 ​× ​10^−19^. (B) Gross morphology of control and *Irgc1* KO testes. (C) Testis weight of control and *Irgc1* KO mice. Average weight of testis was Ctrl ​= ​96.30 ​± ​20.81 ​mg; *Irgc1* KO ​= ​95.54 ​± ​13.37 ​mg. *P* ​= ​0.945. (D) PAS staining of testis and cauda epididymis sections.

To examine whether *Irgc1* is required for male fertility, individual WT or KO males were caged with WT females over a period of three months. During the course of the mating test, KO males showed severe subfertility (Fig. 2A). In order to investigate the cause of subfertility observed in *Irgc1* KO mice, we performed morphological and histological analysis of the testes. First, no significant differences were found in the size and appearance of the testes between the control and *Irgc1* KO mice (Fig. 2B and C). In addition, Periodic acid–Schiff (PAS) staining of testis cross-sections revealed no apparent defects in spermatogenesis (Fig. 2D). Further, PAS staining of the cauda epididymis showed that the tubules were filled with spermatozoa even in *Irgc1* KO mice (Fig. 2D).

### 2.4 IRGC1 is required for sperm tail formation and motility

**Fig. 3:**
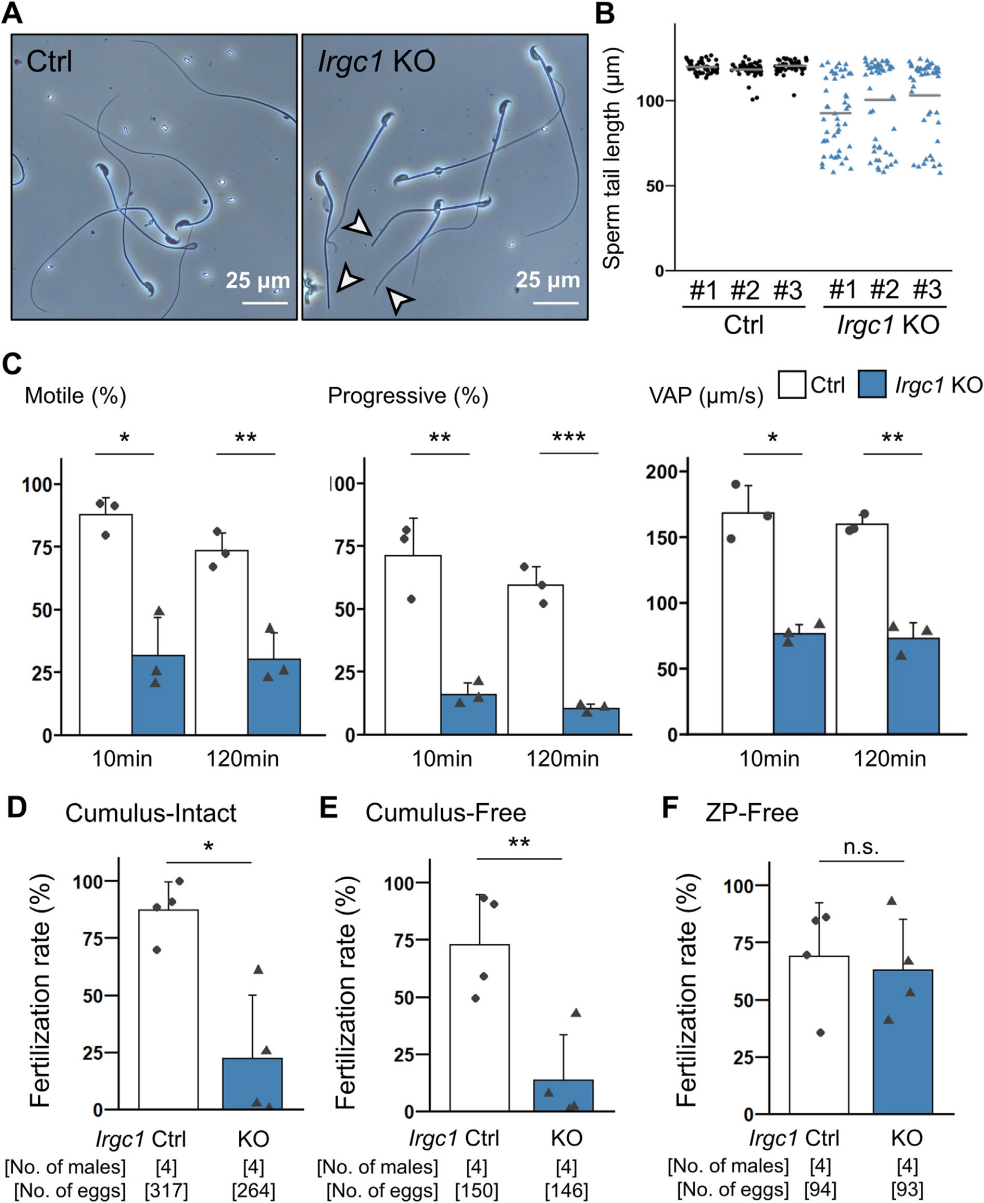
*Irgc1* KO spermatozoa exhibit abnormal tail morphology and impaired motility. (A) Observation of spermatozoa obtained from cauda epididymis. White arrowheads indicate short tails. (B) Analysis of sperm tail length. *Irgc1* KO spermatozoa exhibit short tails. Average length of sperm tail was Ctrl #1 ​= ​119.82 ​± ​3.15 ​μm, Ctrl #2 ​= ​118.28 ​± ​4.44 ​μm, Ctrl #3 ​= ​120.44 ​± ​3.62 ​μm; *Irgc1* KO #1 ​= ​92.65 ​± ​21.46 ​μm, *Irgc1* KO #2 ​= ​100.40 ​± ​25.22 ​μm, *Irgc1* KO #3 ​= ​103.05 ​± ​22.93 ​μm. (C) Sperm motility was analyzed 10 ​min and 120 ​min after incubation in a capacitation medium. Average percentage of motile spermatozoa was Ctrl ​= ​87.57 ​± ​6.91%, *Irgc1* KO ​= ​31.43 ​± ​15.41% for 10 ​min (*P* ​= ​0.013); Ctrl ​= ​73.33 ​± ​7.07%, *Irgc1* KO ​= ​30.03 ​± ​10.54% for 120 ​min (*P* ​= ​0.0062). Percentage of spermatozoa with progressive motility was Ctrl ​= ​70.90 ​± ​14.91%, *Irgc1* KO ​= ​15.70 ​± ​4.54% for 10 ​min (*P* ​= ​0.0036); Ctrl ​= ​59.27 ​± ​7.40%, *Irgc1* KO ​= ​10.10 ​± ​1.71% for 120 ​min (*P* ​= ​0.00036). VAP (average path velocity) was Ctrl ​= ​168.37 ​± ​20.79 ​μm/s, *Irgc1* KO ​= ​76.46 ​± ​6.99 ​μm/s for 10 ​min (*P* ​= ​0.010); Ctrl ​= ​159.79 ​± ​6.98 ​μm/s, *Irgc1* KO ​= ​72.95 ​± ​11.88 ​μm/s for 120 ​min (*P* ​= ​0.0012). (D) IVF analyses using cumulus-intact oocytes. Ctrl ​= ​87.34 ​± ​11.99%; *Irgc1* KO ​= ​22.39 ​± ​27.80%. *P* ​= ​0.012. (E) IVF analyses using cumulus-free oocytes. Ctrl ​= ​72.50 ​± ​21.89%; *Irgc1* KO ​= ​13.31 ​± ​19.97%. *P* ​= ​0.0073. (F) IVF analyses using zona pellucida (ZP)-free oocytes. Ctrl ​= ​68.80 ​± ​23.65%; *Irgc1* KO ​= ​62.54 ​± ​22.23%. *P* ​= ​0.71.

We observed mature spermatozoa collected from the cauda epididymis. Intriguingly, some *Irgc1* KO spermatozoa exhibit short tails (Fig. 3A and B; Fig. S1E). Because no abnormalities were found in the midpiece (Fig. 3A), it is likely that the tips of the tails were shortened. Consistent with abnormal flagellar morphology, we found that the percentage of motile spermatozoa was significantly lower in *Irgc1* KO mice than that of the control mice using computer-assisted sperm analysis (CASA) (Fig. 3C; Movies S1,S2). Further analyses showed that progressive motility and velocity parameters such as average path velocity (VAP), straight line velocity (VSL), and curvilinear velocity (VCL) were significantly impaired in *Irgc1* KO mice after both 10 and 120 ​min incubation in a capacitating medium (Fig. 3C; Figs. S2A and B). These results suggest that abnormal sperm flagellar morphology and motility result in male subfertility.

Supplementary video related to this article can be found at https://doi.org/10.1016/j.ydbio.2022.05.011

The following is/are the supplementary data related to this article:

Multimedia component 5 Multimedia component 5

Multimedia component 6 Multimedia component 6

When we performed *in vitro* fertilization (IVF) using cumulus intact oocytes, fertilization rates were lower in *Irgc1* KO mice (Fig. 3D) consistent with the mating exam (Fig. 2A). Removing cumulus cells could not rescue lower fertilization rates (Fig. 3E); however, when the zona pellucida, an extracellular matrix surrounding the oocyte, was removed, *Irgc1* KO spermatozoa could fertilize oocytes at comparable rates to the control spermatozoa (Fig. 3F). These results suggest that *Irgc1* KO spermatozoa can undergo the acrosome reaction and fuse with eggs, but cannot penetrate the zona pellucida likely due to impaired sperm motility.

### 2.5 IRGC1 is important for the FS formation

**Fig. 4:**
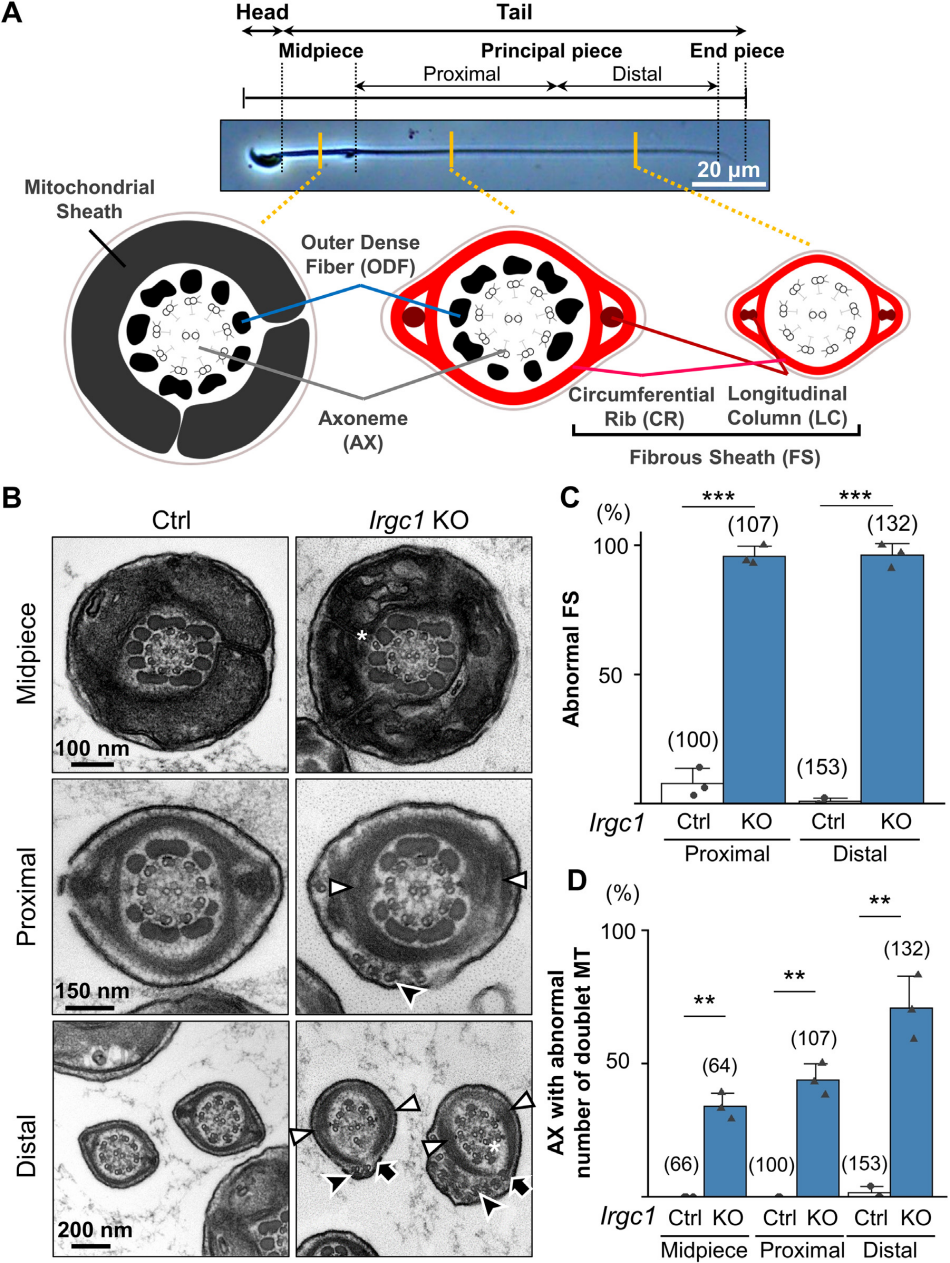
*Irgc1* KO spermatozoa exhibit abnormal FS structures. (A) Schematic drawing of the internal structures of mouse spermatozoa. Proximal and distal regions of the principal piece were defined as having 5–9 and 0–4 outer dense fibers, respectively. (B) Cross-sections of the midpiece and principal piece (proximal and distal regions). Extra microtubules are indicated by white asterisks. Disruption of LCs is indicated by white arrowheads. Torn FS is indicated by black arrows. Doublet microtubules located outside of the FS are indicated by black arrowheads. (C) Quantification of the morphological analysis of FS. The number of flagellar sections analyzed are shown above each bar. Average percentage of abnormal FS was Ctrl ​= ​7.82 ​± ​5.79%, *Irgc1* KO ​= ​95.77 ​± ​3.70% for the proximal region (*P* ​= ​8.5 ​× ​10^−5^); Ctrl ​= ​0.62 ​± ​1.07%, *Irgc1* KO ​= ​96.06 ​± ​4.40% for the distal region (*P* ​= ​0.00038). (D) Quantification of axonemal structures (AX) with abnormal number of doublet microtubules (MTs). The number of flagellar sections analyzed are shown above each bar. Average percentage of AX with abnormal number of doublet MTs was Ctrl ​= ​0.00 ​± ​0.00%, *Irgc1* KO ​= ​33.96 ​± ​4.89% for the midpiece (*P* ​= ​0.0068); Ctrl ​= ​0.00 ​± ​0.00%, *Irgc1* KO ​= ​43.70 ​± ​6.05% for the proximal region (*P* ​= ​0.0063); Ctrl ​= ​1.36 ​± ​2.36%, *Irgc1* KO ​= ​70.76 ​± ​12.02% for the distal region (*P* ​= ​0.0080).

Because the short tails observed in *Irgc1* KO spermatozoa are similar to *Akap3* or *Akap4* KO spermatozoa that exhibit not only short tails but also abnormal FS morphology (Xu et al., 2020; Miki et al., 2002), we observed the ultrastructure of the *Irgc1* KO spermatozoa (Fig. 4A) using transmission electron microscopy (TEM). No differences were found in the mitochondrial sheath of the midpiece between the control and *Irgc1* KO spermatozoa (Fig. 4B). In contrast, the cross-sections of principal pieces revealed that *Irgc1* KO spermatozoa possessed disorganized FS (Fig. 4B and C; Fig. S3A). The longitudinal columns (LCs) that are localized adjacent to the doublet microtubules 3 and 8 were not thickened in *Irgc1* KO spermatozoa (Fig. 4B). Further, the ribs that circumferentially connect LCs were torn (Fig. 4B). Besides the FS structure, abnormal numbers of microtubule doublets were observed in both midpiece and principal piece of *Irgc1* KO spermatozoa (Fig. 4B,D; Figs. S3B,C,D) and even some KO spermatozoa exhibit the microtubule doublets outside of the FS (Fig. 4B). These abnormalities in microtubule doublets were more frequently observed in the distal part of the principal piece rather than the midpiece and the proximal part of the principal piece (Fig. 4D; Fig. S3E).

During spermatogenesis, LCs are formed before the ribs (Eddy et al., 2003). To examine whether the defects in the FS of *Irgc1* KO spermatozoa are due to abnormalities in initial LC formation, we examined testicular spermatozoa using TEM. Cross-sections of step 9 *Irgc1* KO spermatids revealed that LCs were formed correctly (Fig. S4), suggesting that the initial formation of the LCs was not disrupted. In contrast, thin LCs and torn ribs were observed in step 16 spermatids of *Irgc1* KO mice, indicating that the FS formation was disrupted in later stages of spermatogenesis. Further, no abnormalities were found in the axoneme at step 9 or 16 (Fig. S4), suggesting that the displacement of the axoneme occurs at the very late stages of spermatogenesis or in the epididymis. Taken together, these results indicate that *Irgc1* KO spermatozoa exhibit abnormal tail morphology and motility likely due to impaired FS formation.

### 2.6 IRGC1 is localized in the sperm tail

**Fig. 5:**
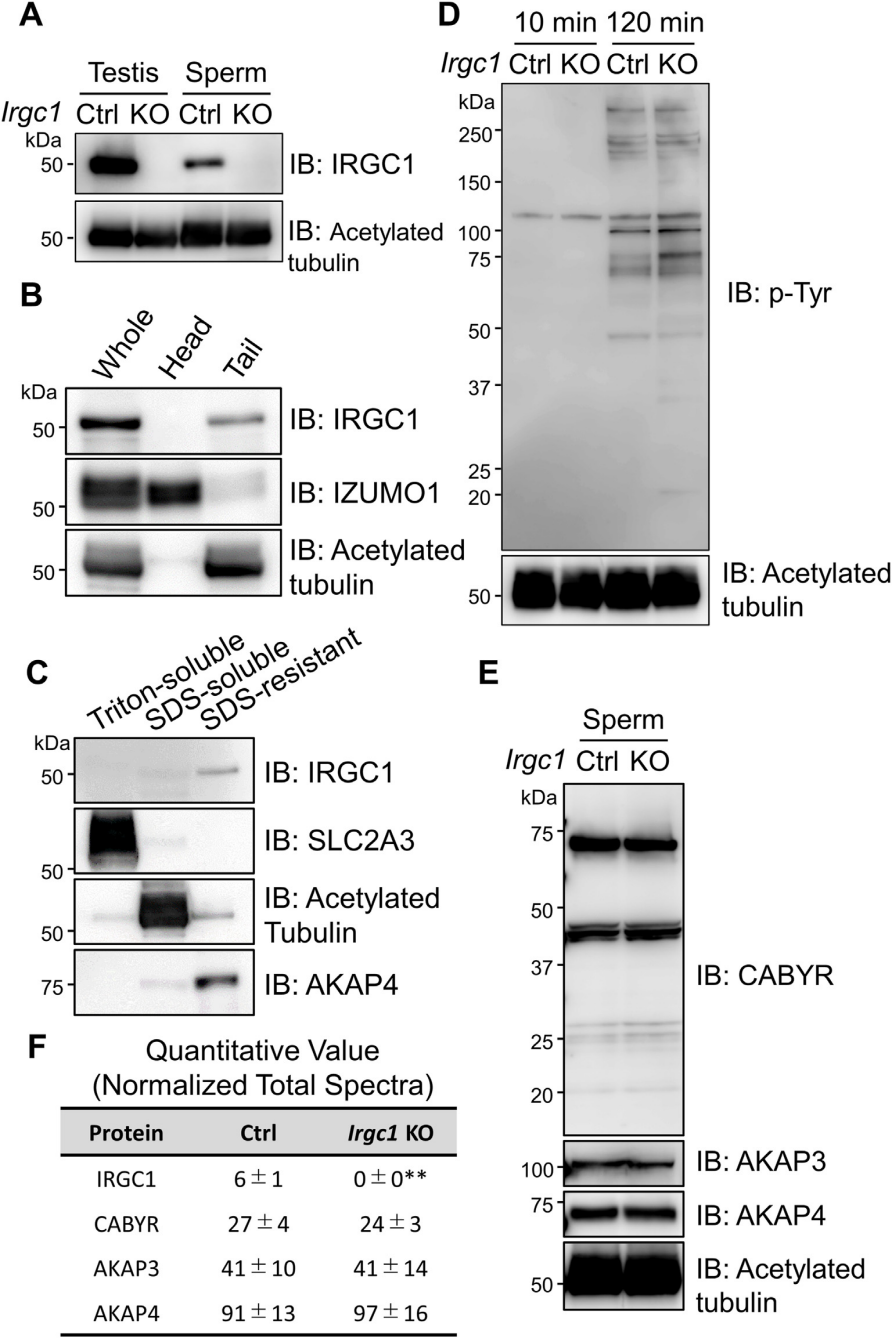
IRGC1 is localized in the sperm tail. (A) IRGC1 was detected in the testis and cauda epididymal spermatozoa in the control, but not in *Irgc1* KO mice. Acetylated tubulin as a loading control. Representative images of three experiments are shown. (B) Head and tail separation of mouse spermatozoa. IRGC1 is found in the tail fraction. IZUMO1 and acetylated tubulin were used as a marker for heads and tails, respectively. The experiment was done in duplicate. (C) Fractionation of the spermatozoa using different lysis buffers. IRGC1 was found in the SDS-resistant fraction that contains FS proteins. SLC2A3, acetylated tubulin, and AKAP4 were used as a marker for Triton-soluble, SDS-soluble, and SDS-resistant fraction, respectively. The experiment was done in duplicate. (D) Immunoblotting analysis of phosphorylated tyrosine residues (p-Tyr) in spermatozoa. Spermatozoa were incubated in a capacitation medium for 10 ​min and 120 ​min. Acetylated tubulin as a loading control. The experiment was done in duplicate. (E) No differences in the amounts of FS proteins were observed between control and *Irgc1* KO spermatozoa. Acetylated tubulin as a loading control. (F) MS analysis of SDS-resistant fraction. IRGC1 was detected in the SDS-resistant fraction of the control mice, but not in *Irgc1* KO mice. No differences were found in other FS proteins. Number of males ​= ​3 for the control and 4 for *Irgc1* KO.

We performed immunoblotting analysis to confirm the deletion of IRGC1 in *Irgc1* KO mice (Fig. 5A). A single band of approximately 50 ​kDa was detected in the control but was absent in the KO lysates, suggesting that the antibody works for immunoblotting. To analyze the localization of IRGC1 in mature spermatozoa, we tried immunofluorescence, but the antibody did not work. We then separated sperm heads and tails for immunoblotting analysis and detected the IRGC1 band in the tail fraction (Fig. 5B). Further, we performed a sperm fractionation assay as described previously (Cao et al., 2006; Castaneda et al., 2017) and found that IRGC1 is localized in the SDS-resistant fraction that contains FS and ODF proteins including AKAP4 (Fig. 5C). Considering that *Irgc1* KO spermatozoa exhibit abnormal FS structures, these results suggest that IRGC1 is a component of the FS.

During sperm incubation in a capacitation medium, several sperm proteins are tyrosine-phosphorylated (Visconti et al., 1995). Because tyrosine-phosphorylated proteins are predominantly localized in the FS (Zhao and Kan, 2019), we analyzed the tyrosine-phosphorylation status, but no differences were found between the control and *Irgc1* KO spermatozoa (Fig. 5D). To determine whether the absence of IRGC1 affects the amount of other FS proteins, we performed immunoblotting analysis of AKAP3, AKAP4 and CABYR (Xu et al., 2020; Miki et al., 2002; Young et al., 2016), but no differences were observed (Fig. 5E). Further, we performed mass spectral analysis of the SDS-resistant fraction. Consistent with the immunoblotting result (Fig. 5C), we detected IRGC1 in the SDS-resistant fraction in the control, but not in the KO (Fig. 5F; Tables S1 and S2). No apparent differences were detected in other FS proteins including AkAP3, AKAP4, and CABYR. These results suggest that IRGC1 is not involved in the tyrosine-phosphorylation status or localization of major known FS components.

## 3 Discussion

IRGC1 was identified as a member of immunity-related GTPases, but its function was unclear especially because its expression was not induced by interferons (IFNs) (Bekpen et al., 2005). In this study, we knocked out *Irgc1* in mice using the CRISPR/Cas9 system and found that *Irgc1* KO males showed reduced fertility. Further analyses revealed that IRGC1 is important for normal sperm motility. Although it remains to be determined if IRGC1 plays direct roles in regulating sperm motility in mature spermatozoa, our results suggest that sperm motility is impaired due to abnormal FS formation in the testis. These results indicate that the function of IRGC1 is different from other IRGs that play roles in pathogen resistance.

Our EM analyses revealed that the formation of not only LCs and ribs but also the axoneme is disrupted in *Irgc1* KO mice. Because disrupted axonemes were also found in *Cabyr* or *Eno4* KO mice that exhibit impaired FS formation (Young et al., 2016; Nakamura et al., 2013), the abnormal axonemal structures can be a secondary effect of disrupted FS. This idea is supported by the fact that IRGC1 was detected in the SDS-resistant fraction that contains FS proteins, but not in the SDS-soluble fraction that contains axonemal proteins. Because the axoneme structures were normal at step 9 and 16 of spermiogenesis (Fig. S4), the displacement of the axoneme occurs after its formation likely due to abnormal FS structures. Because it is unlikely that extra doublet microtubules are generated de novo, the axoneme may be disrupted due to abnormal microtubule sliding, which could result in the axoneme with less or more microtubules in the cross section. Microtubule abnormalities were more frequently observed in the distal part of the principal piece (Fig. 4D; Fig. S3E). Because the FS is thinner and the number of ODF is less in the distal region, the axoneme structure may be less stabilized in this region. Disruption of the axoneme after its formation has also been reported in KO mice of other genes such as *Dnah17* (Zhang et al., 2020) and *Cfap97d1* (Oura et al., 2020), and more frequent microtubule abnormalities in the distal region were found in *Dnah17* KO mice as well.

Involvement of IRGC1 in the FS formation is also supported by its conservation among species. For example, by searching the National Center for Biotechnology Information (NCBI) database, IRGC1 was not found in zebrafish or *Xenopus* whose spermatozoa have an axoneme but not FS although other IRGs were found in these species. In contrast, the number of IRGs were diminished or reduced in birds and humans likely due to dispensable function of IRGs in pathogen resistance in these species; however, IRGC1 is conserved well in these species whose spermatozoa possess FS. IRGs were found even in cephalochordates and suggested to play conserved immune functions in vertebrates and cephalochordates (Li et al., 2009). IRGC1 may be evolved later as spermatozoa acquire the FS in species such as reptiles, birds, and mammals.

AKAP3, AKAP4, and CABYR have been identified as proteins that comprise the FS. Absence of these proteins in spermatozoa results in disruption of the FS. For example, *Akap4* KO spermatozoa show disrupted structures of both LCs and ribs (Miki et al., 2002). *Akap3* KO spermatozoa show lack of ribs but not LCs (Xu et al., 2020). Further, *Cabyr* KO spermatozoa show abnormalities preferentially in ribs (Young et al., 2016). Recent report suggested that *Akap3* or *Akap4* KO mice exhibited a global change in the sperm proteome, especially, in proteins that are related to the FS (Xu et al., 2020). For example, both *Akap3* and *Akap4* KO spermatozoa exhibited significant decrease of ropporin 1 (ROPN1), ropporin 1-like (ROPN1L), and sperm autoantigenic protein 17 (SPA17) (Xu et al., 2020). However, we did not find significant differences in the amount of these proteins between the control and *Irgc1* KO mice (Table S1). Consistent with fewer impacts on the proteome of the FS, the disruption of the FS is milder in *Irgc1* KO mice than that of *Akap3* or *Akap4* KO mice. These results indicate that IRGC1 may play roles in fine-tuning the FS formation. Because our fractionation assay detected IGRC1in the fraction of FS proteins (Fig. 5C), IRGC1 may be a structural component of the FS. IRGC1 has a GTP-binding motif like other IRGs and analysis of this domain may lead to further functional elucidation of IRGC1.

In summary, our results reveal that IRGC1 is important for the formation of the FS, a unique accessory structure of the spermatozoa, which may give insight into the molecular evolution of IRGC1 that is apart from other IRGs. *IRGC1* is conserved well in humans (Fig. S1A) and a variation in IRGC1 that causes a frameshift (p.Leu14AlafsTer19) was found (variant ID: 19-44222636-CCT-C, allele frequency: 0.0002060) (gnomAD). Further analysis of IRGC1 may provide a better understanding of the mechanism that regulates the FS formation and may lead to better treatment for male infertility, especially a dysplasia of the fibrous sheath.

## 4 Materials and methods

### 4.1 Animals

All animal experiments were approved by the Animal Care and Use Committee of the Research Institute for Microbial Diseases, Osaka University. Mice were purchased from CLEA Japan (Tokyo, Japan) or Japan SLC (Shizuoka, Japan). WT or *Irgc1* heterozygous mice were used as controls.

### 4.2 Phylogenetic analysis

IRGC1 paralogues in *Mus musculus* were identified using Protein Blast (https://blast.ncbi.nlm.nih.gov/Blast.cgi) and the proteins with more than 30% identity are listed. Phylogenetic analysis was performed using the Neighbor-Joining method with MEGA X (Kumar et al., 2018). The evolutionary distances (the units of the number of amino acid differences per site) were computed using the p-distance method (Nei and Kumar, 2000).

### 4.3 Isolation of RNA and RT-PCR

Mouse adult multi-tissues and mouse testes at different ages were obtained from C57BL/6N mice. RNA samples were isolated and purified using TRIzol (Thermo Fisher Scientific, Waltham, MA, USA). RNA was reverse transcribed to cDNA using SuperScript IV First-Strand Synthesis System (Thermo Fisher Scientific) using an oligo (dT) primer. PCR was performed using primers that are listed in Table S3.

### 4.4 *In silico* expression data analysis

Single-cell transcriptome data in the mouse and human testis was obtained (Hermann et al., 2018). *Irgc1* expression in those cells was analyzed using Loupe Cell Browser 3.3.1 (10X Genomics, Pleasanton, CA, USA).

### 4.5 Generation of *Irgc1* KO mice using the CRISPR/Cas9 system

KO mice were generated as described previously (Abbasi et al., 2018). The gRNAs with fewer off-target sites were designed utilizing the online source CRISPRdirect (Naito et al., 2015). The gRNA target sequences were 5′- AGACTCAAAAGCAGTGCGCA -3′ and 5′- TTAGTGGAAAAGCGGAGCAC -3′. KO mice were maintained on a B6D2 background. *Irgc1* mutant mice have been assigned labels (B6D2-Irgc1<em1Osb>) and deposited into the RIKEN BioResource Research Center (ID#: RBRC11221) and the Center for Animal Resources and Development (CARD), Kumamoto University (ID#: 3025).

### 4.6 Fertility test

For the *in vivo* fertility analysis, sexually mature KO male mice or control male mice were caged with three 8-week-old B6D2F1 female mice for three months and plugs were checked every morning. The number of pups was counted on the day of birth. For the *in vitro* fertility assay, *in vitro* fertilization (IVF) analysis was performed as previously described (Morohoshi et al., 2021) with some minor changes. For ZP-free oocytes, sperm insemination was performed at a final density of 2 ​× ​10^4^ spermatozoa/mL.

### 4.7 Histological analysis

PAS staining of sections were performed as previously described (Morohoshi et al., 2020). Testes or cauda epididymis were fixed at 4 ​°C in Bouin's solution (Polysciences, Inc., Warrington, PA, USA) and were processed for paraffin embedding. Paraffin sections were cut at a thickness of 5 ​μm using a HM325 microtome (Microm, Walldorf, Germany). After rehydrating the sections, they were stained with 1% periodic acid (Nacalai Tesque, Kyoto, Japan) and Schiff's reagent (FUJIFILM WakoPure Chemical, Osaka, Japan) for 20 ​min each at room temperature. The sections were then counterstained with Mayer hematoxylin solution (FUJIFILM WakoPure Chemical). The sections were observed with an Olympus BX-53 microscope (Tokyo, Japan).

### 4.8 Morphological and motility analysis of spermatozoa

Spermatozoa extracted from cauda epididymis were suspended in TYH medium (Muro et al., 2016). After 10 ​min incubation, spermatozoa were collected to observe morphology. Sperm motility was analyzed as previously described (Miyata et al., 2020, 2021). The motility of more than 200 spermatozoa was measured after incubation at 10 ​min and 120 ​min in TYH medium using CEROS II (software version 1.4; Hamilton Thorne Biosciences, Beverly, MA, USA). For movies, sperm motility was observed using an Olympus BX-53 microscope equipped with a high-speed camera (HAS-L1, Ditect, Tokyo, Japan).

### 4.9 Protein extraction from testes and spermatozoa

Testes or spermatozoa were homogenized in sample buffer containing 1 ​M Tris-HCL pH 6.8, 2% SDS, 10% Glycerol, and 0.005% Bromophenol Blue, and boiled for 5 ​min. Supernatants were obtained after centrifugation at 15,300×*g* for 15 ​min at 4 ​°C.

### 4.10 Immunoblot analysis

Immunoblot analysis was performed as described previously (Young et al., 2016). Samples were subjected to sodium dodecyl sulphate polyacrylamide gel electrophoresis (SDS-PAGE) under reduced conditions using 2-ME before transferred onto a polyvinylidene difluoride membrane. For phosphorylated tyrosine, anti-phosphorylated-tyrosine antibody clone 4G10 (1/1000, 05–321, Merck Millipore, Burlington, MA, USA); for IRGC1, anti-IRGC1 polyclonal antibody (1/1000, 14090-1-AP, ProteinTech, Rosemont, IL, USA); for AKAP3, anti-AKAP3 polyclonal antibody (1/1000, 13907-1-AP, ProteinTech); for AKAP4, anti-AKAP82 monoclonal antibody (1/1000, 611564, BD Transduction Laboratories, Franklin Lakes, NJ, USA); for SLC2A3, anti-SLC2A3 monoclonal antibody (1:1000, KS63-10, Fujihara et al., 2012); for IZUMO1, anti-IZUMO1 monoclonal antibody (1:1000, KS64-125, Ikawa et al., 2011); for acetylated tubulin, anti-acetylated tubulin monoclonal antibody (1:5,000, T7451, Sigma-Aldrich, St. Louis, MO, USA) were used. Immunoreactive proteins were detected using Chemi-Lumi One Super (Nacalai Tesque) or Chemi-Lumi One Ultra (Nacalai Tesque).

### 4.11 Sperm head-tail separation

Sperm head-tail separation was performed as previously described (Miyata et al., 2015) with some minor changes. Spermatozoa obtained from the cauda epididymis were suspended in 1 ​mL PBS and sonicated to separate tails from heads on ice (Sonifier SLPe, Branson Ultrasonics, Brookfield, CT, USA). The sample was centrifuged at 15,000×*g* for 15 ​min. The pellet was then resuspended with 200 ​μL PBS and mixed with 1.8 ​mL of 90% Percoll solution (GE Healthcare, Chicago, IL, USA) in PBS. After centrifugation at 15,000×*g* for 15 ​min, sperm heads were in the bottom of the tube and the tails were in the top layer of the solution. The separated sample was diluted with five-fold PBS and centrifuged at 10,000×*g* for 15 ​min. The pellets were washed twice with PBS and dissolved in sample buffer.

### 4.12 Fractionation of spermatozoa

Fractionation of spermatozoa obtained from the cauda epididymis was performed as described previously (Cao et al., 2006; Castaneda et al., 2017).

### 4.13 Mass spectral analysis of the SDS-resistant fraction

Spermatozoa from the cauda epididymis were collected and washed in PBS. In order to enrich the FS proteins, fractionation of spermatozoa was conducted as previously described (Cao et al., 2006; Castaneda et al., 2017). After the SDS-soluble fraction was removed, the pellet was dissolved in sample buffer and boiled for 5 ​min. LC-MS/MS was performed by the BIKEN mass spectrometry core at Osaka University, Japan. UniProt mouse database was used to match the detected proteins. Exponentially modified protein abundance index (emPAI), which enables us to estimate relative quantitation of proteins in a mixture (Ishihama et al., 2005), was calculated using Mascot (version: 2.7.0, Matrix Science, London, UK).

### 4.14 Observation of sperm flagellum ultrastructure using TEM

The sections of testes and cauda epididymis were prepared as previously described (Shimada et al., 2019). The prepared sections were observed using a JEM-1400 plus electron microscope (JEOL, Tokyo, Japan) at 80 ​kV with a CCD Veleta 2 ​K ​× ​2 ​K camera (Olympus).

### 4.15 Statistical analysis

Statistical analyses were carried out using the two-tailed Welch's *t*-test using Microsoft Office Excel 2016 (Microsoft Corporation, Redmond, WA, USA). Differences were considered significant at P ​< ​0.05 (∗), P ​< ​0.01 (∗∗), P ​< ​0.001 (∗∗∗). Error bars are standard deviation.

## Funding

This research was supported by the 10.13039/501100001700Ministry of Education, Culture, Sports, Science and Technology (MEXT)/10.13039/501100001691Japan Society for the Promotion of Science (10.13039/501100001691JSPS) KAKENHI grants (JP21K19569, JP22H03214 to H.M., JP20K16107 to K.S., and JP19H05750, JP21H04753, JP21H05033 to M.I.); 10.13039/100007449Takeda Science Foundation grant to H.M., K.S., and M.I.; the 10.13039/100009619Japan Agency for Medical Research and Development (AMED) grant (JP21gm5010001 to M.I.); JST FOREST (JPMJFR211F to H.M.); the 10.13039/100009633Eunice Kennedy Shriver National Institute of Child Health and Human Development (P01HD087157 and R01HD088412 to M.I.); and the 10.13039/100000865Bill & Melinda Gates Foundation (Grand Challenges Explorations grant INV-001902 to M.I.).

## Declaration of competing interest

The authors declare no competing financial interests.
