# Supplementary material for: IRGC1, a testis-enriched immunity related GTPase, is important for fibrous sheath integrity and sperm motility in mice
Source: Dev Biol. 2022 Aug;488:104–13. doi: 10.1016/j.ydbio.2022.05.011 (PMC9232189; doi:10.1016/j.ydbio.2022.05.011)
Supplement: Multimedia component 2 [file mmc2.docx]

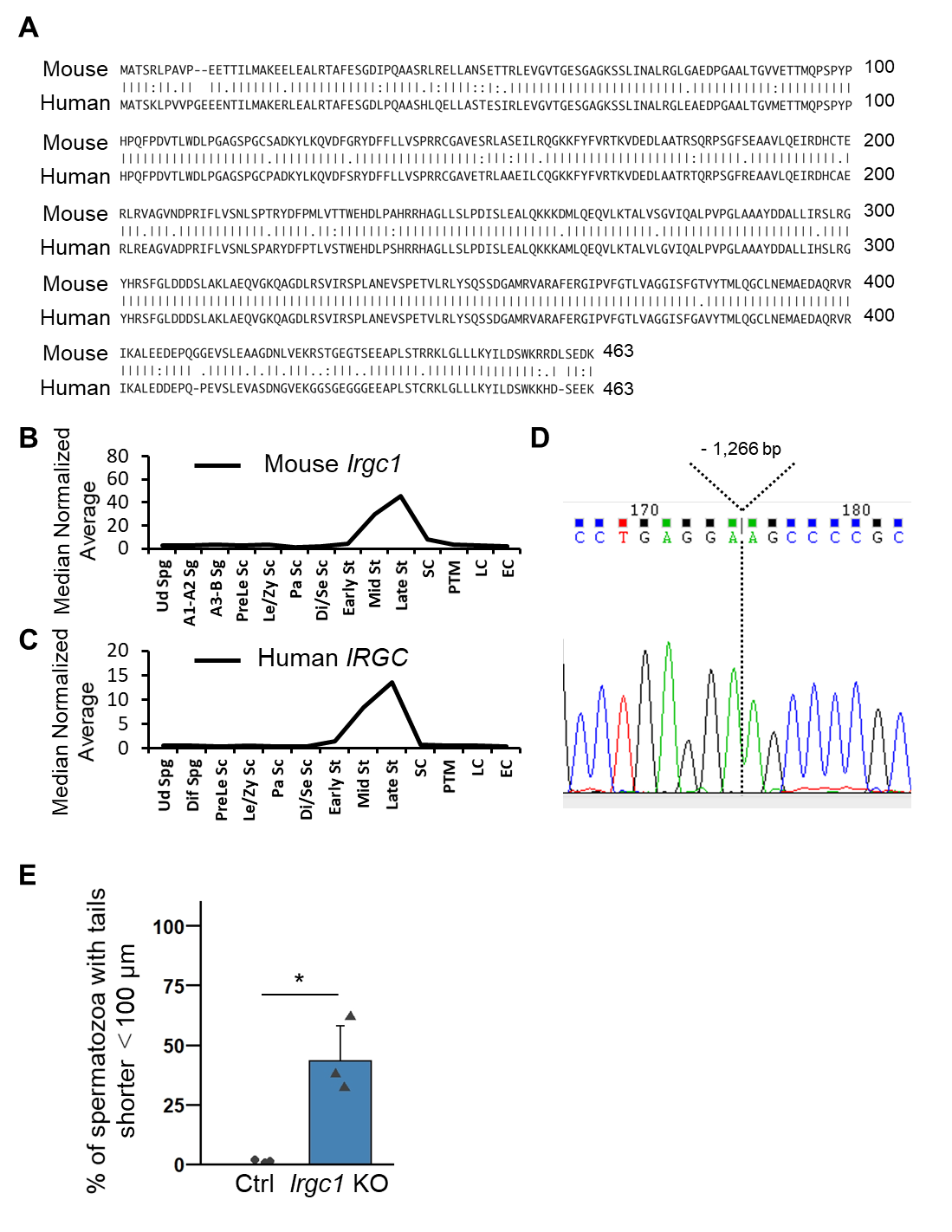


**Fig. S1. Expression pattern of mouse *Irgc1* and human *IRGC*.**

(A) Amino acid sequences of mouse IRGC1 and human IRGC. Pairwise sequence alignment was performed using EMBOSS Needle [(https://www.ebi.ac.uk/Tools/psa/emboss_needle/](file:///C:/Users/Miyata/Desktop/(https:/www.ebi.ac.uk/Tools/psa/emboss_needle/)). Pairs of identical, highly conserved, semi-conserved, and non-conserved amino acids are indicated by black lines, colons, periods, no lines, respectively. (B) and (C) *in silico* expression data analysis of mouse *Irgc1* and human *IRGC* in testis. Ud spg: Undifferentiated spermatogonia, A1- A2 Sg: A1 and A2 differentiating spermatogonia, A3-B Sg: A3, A4, In, and B differentiating spermatogonia, Dif Spg: differentiating spermatogonia, PreLe Sc: preleptotene spermatocytes, Le/Zy Sc: leptotene/zygotene spermatocytes, Pa Sc: pachytene spermatocytes, Di/Se Sc: diplotene/secondary spermatocytes, Early St: early round spermatids, Mid St: mid round spermatids, Late St: late round spermatids, SC: Sertoli cells, PTM: peritubular myoid cells, LC: Leydig cells, and EC: Endothelial cells. (D) Wave pattern sequence of *Irgc1* KO mice. *Irgc1* KO allele exhibits a 1,266 bp deletion. (E) Percentages of spermatozoa with tails shorter than 100 μm. The percentages were calculated from Fig. 3B. Ctrl = 0.00±0.00%, *Irgc1* KO = 43.33±14.74 % (*P* = 0.036).

**
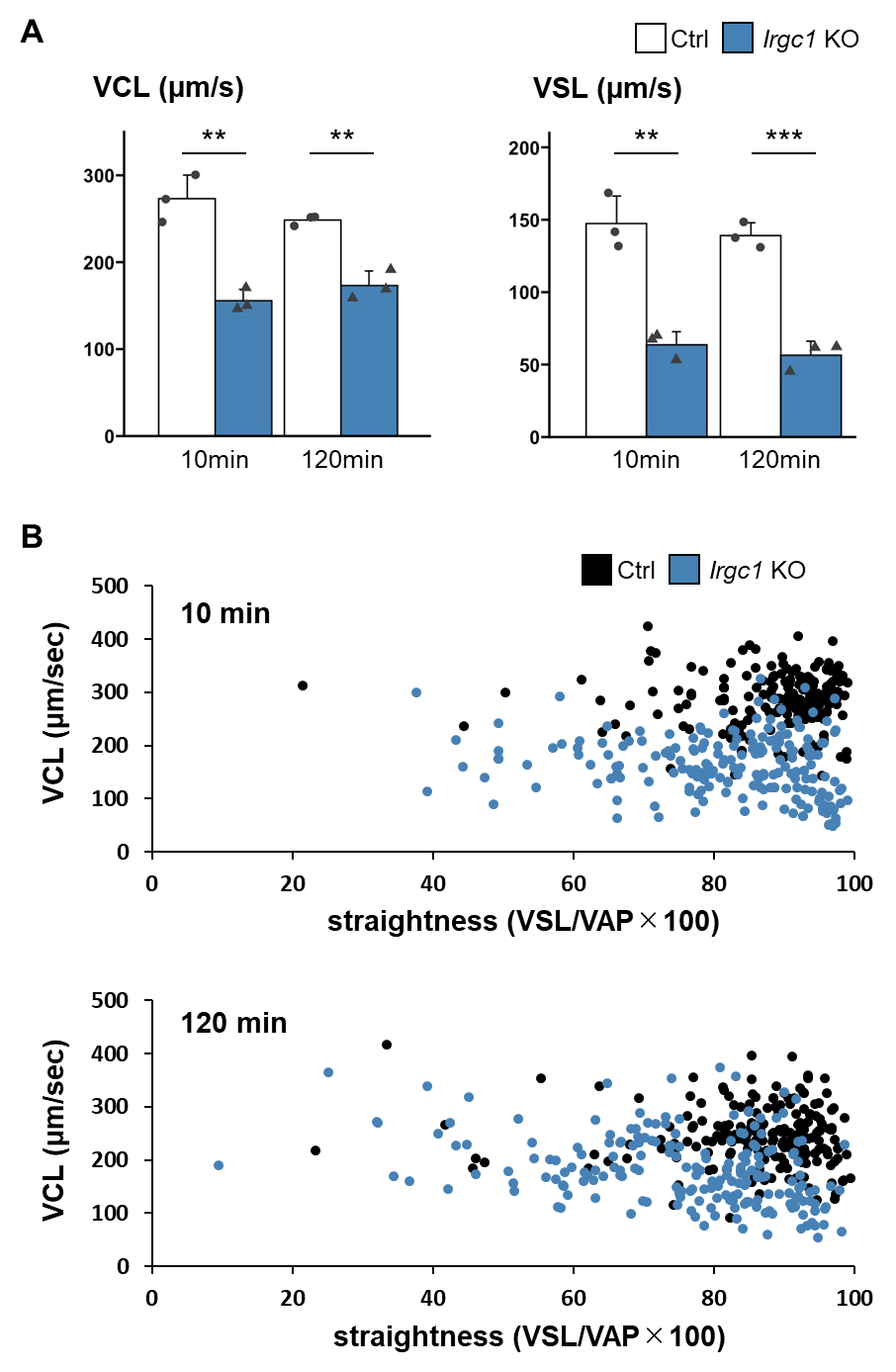
**

**Fig. S2. Motility analysis of *Irgc1* KO spermatozoa.**

(A) Sperm motility was analyzed 10 min and 120 min after incubation in a capacitation medium. VCL (curvilinear velocity) was Ctrl = 273.12±27.15 µm/s, *Irgc1* KO = 155.72±13.02 µm/s for 10 min (*P* = 0.0076); Ctrl = 248.49±5.82 µm/s, *Irgc1* KO = 173.12±16.74 µm/s for 120 min (*P* = 0.0096). VSL (straight-line velocity) was Ctrl = 147.36±19.00 µm/s, *Irgc1* KO = 63.64±9.07 µm/s for 10 min (*P* = 0.0073); Ctrl = 139.05±8.83 µm/s, *Irgc1* KO = 56.47±9.69 µm/s for 120 min (*P* = 0.00042). (B) VCL and straightness (VSL/VAP x 100) were plotted for each spermatozoon. Fifty spermatozoa from 4 males each were analyzed. There are some spermatozoa with high VCL even in *Irgc1* KO mice.


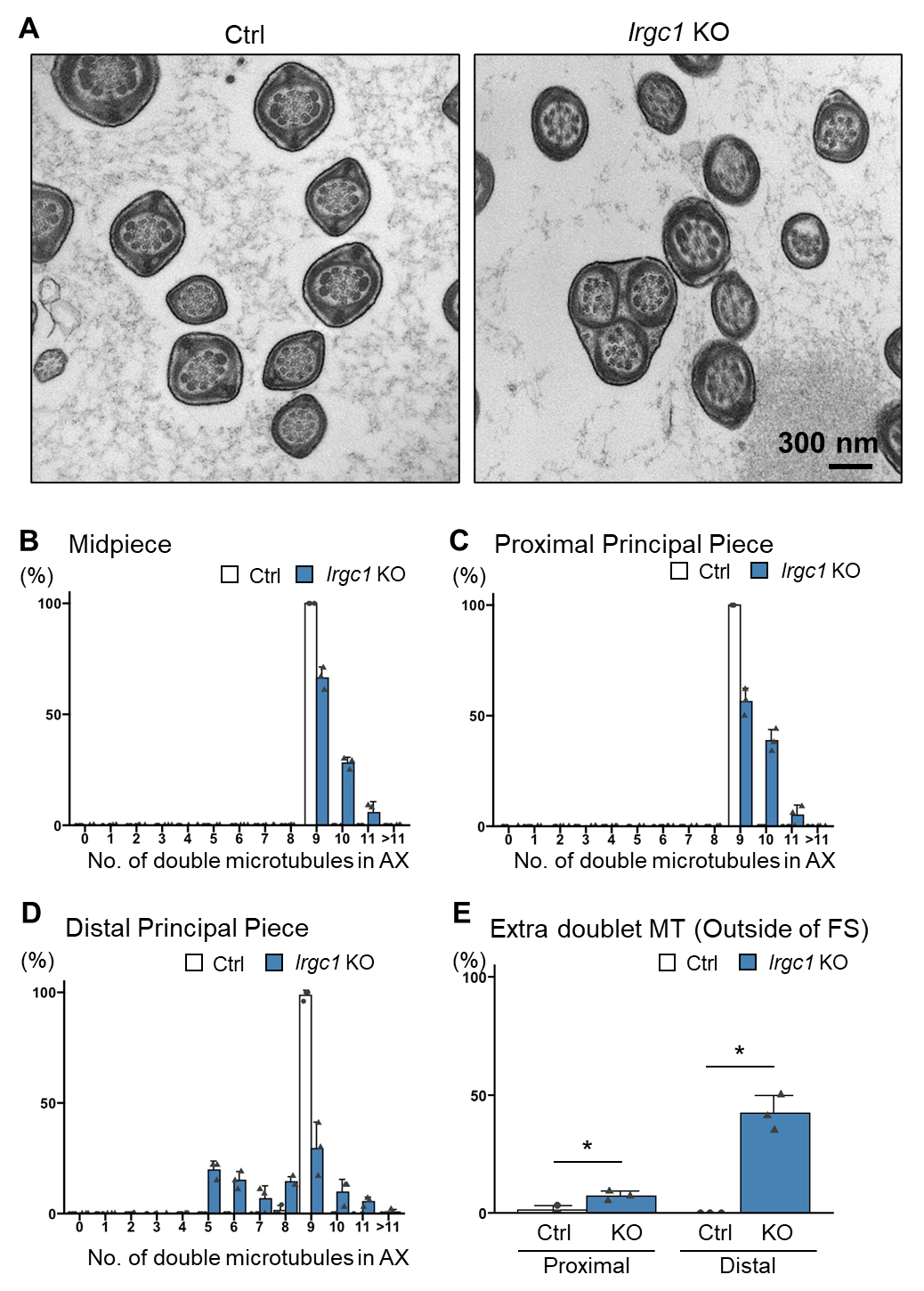


**Fig. S3. Ultrastructural analysis of FS and doublet microtubules in the cauda epididymis.**

(A) Cross-sections of principal pieces of mature spermatozoa in cauda epididymis. Abnormal FS and axonemes were observed in *Irgc1* KO mature spermatozoa. (B)-(E) Quantification of doublet microtubules in midpiece (B), proximal principal piece (C), and distal principal piece (D). Number of double microtubules were counted. (E) Quantification of sections with extra doublet microtubules outside of FS. Average percentage was Ctrl = 1.04±1.80%, *Irgc1* KO = 6.76±2.14% for the proximal region (*P* = 0.025); Ctrl = 0.00±0.00%, *Irgc1* KO = 41.84$\pm$7.67% for the distal region (*P* = 0.011).


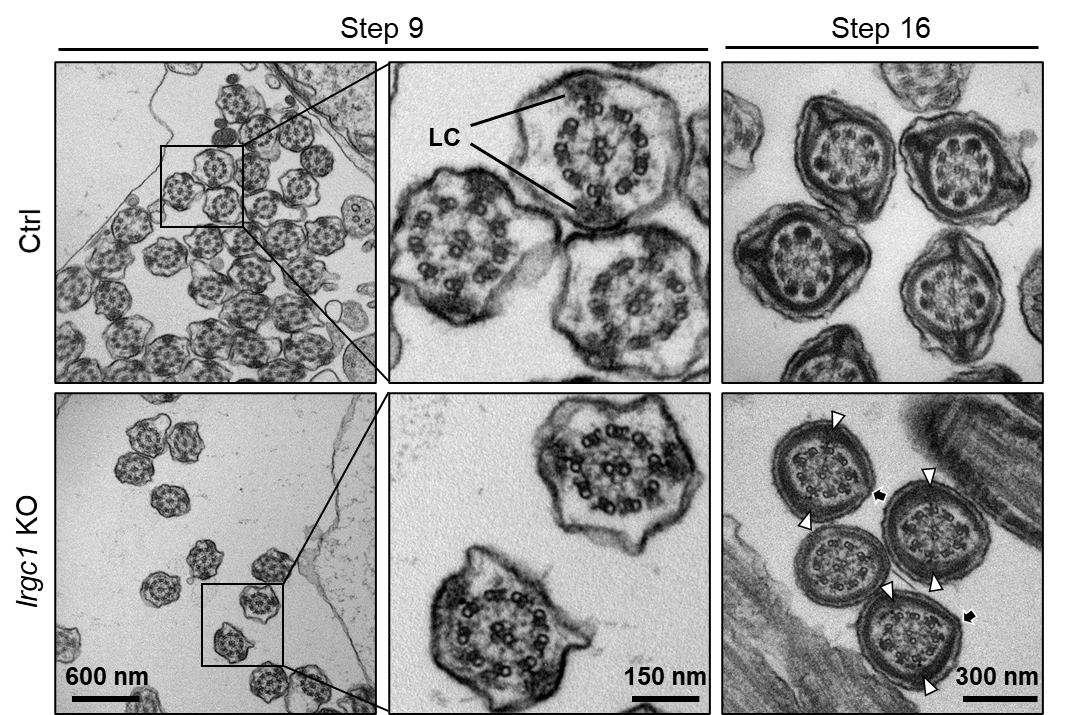


**Fig. S4. Ultrastructural analysis of FS and doublet microtubules in the testis.**

Cross-sections of elongating spermatids in seminiferous tubules. No disruption of LCs or axonemes was observed in step 9 spermatids. In step 16 spermatids, no disrupted axonemal structures were observed, but the FS was disrupted. Thin LCs are indicated by white arrowheads. Torn FS is indicated by black arrows.

**Table S1. Mass spectrometry analysis of sperm SDS-resistant fraction (total spectra).**

Quantitative value normalized total spectra was shown.

**Table S2. Mass spectrometry analysis of sperm SDS-resistant fraction (emPAI).**

Quantitative value normalized emPAI was shown.

**Table S3. Primers used in this study.**

**Movie S1. Sperm motility of *Irgc1* heterozygous male.**

Ten min incubation in TYH medium. Movie is recorded at 50 frames per second (fps) and played at 25 fps (1/2 speed).

**Movie S2. Sperm motility of *Irgc1* KO male.**

Ten min incubation in TYH medium. Movie is recorded at 50 frames per second (fps) and played at 25 fps (1/2 speed).
